# Hepatitis B Virus Infection in HIV-Positive Individuals in the UK Collaborative HIV Cohort (UK CHIC) Study

**DOI:** 10.1371/journal.pone.0049314

**Published:** 2012-11-07

**Authors:** Huw Price, Loveleen Bansi, Caroline A. Sabin, Sanjay Bhagani, Andrew Burroughs, David Chadwick, David Dunn, Martin Fisher, Janice Main, Mark Nelson, Deenan Pillay, Alison Rodger, Chris Taylor, Richard Gilson, UK Collaborative HIV Cohort Hepatitis Group, Steering Committee

**Affiliations:** 1 Centre for Sexual Health and HIV Research, Research Department of Infection and Population Health, University College London, London, United Kingdom; 2 HIV Epidemiology and Biostatistics Group, Research Department of Infection and Population Health, University College London, London, United Kingdom; 3 The Department of Infectious Diseases and HIV Medicine, Royal Free Hospital, London, United Kingdom; 4 Royal Free Hospital and University College London, London, United Kingdom; 5 Department of Infection and Travel Medicine, The James Cook University Hospital, Middlesbrough, United Kingdom; 6 Medical Research Council Clinical Trials Unit, London, United Kingdom; 7 Department of Sexual Health and HIV, Royal Sussex County Hospital, Brighton, United Kingdom; 8 Department of Medicine, Imperial College, London, United Kingdom; 9 HIV and Sexual Health Services, Chelsea and Westminster Hospital, London, United Kingdom; 10 Research Department of Infection, University College London, London, United Kingdom; 11 Research Department of Infection and Population Health, University College London, London, United Kingdom; 12 Caldecot Centre, King’s College Hospital, London, United Kingdom; Saint Louis University, United States of America

## Abstract

**Background:**

Hepatitis B virus (HBV) infection is an increasingly important cause of morbidity and mortality in HIV-infected adults. This study aimed to determine the prevalence and incidence of HBV in the UK CHIC Study, a multicentre observational cohort.

**Methods and Findings:**

12 HIV treatment centres were included. Of 37,331 patients, 27,450 had at least one test (HBsAg, anti-HBs or anti-HBc) result post-1996 available. 16,043 were white, 8,130 black and 3,277 other ethnicity. Route of exposure was homosexual sex 15,223 males, heterosexual sex 3,258 males and 5,384 females, injecting drug use 862 and other 2,723. The main outcome measures used were the cumulative prevalence and the incidence of HBV coinfection. HBV susceptible patients were followed up until HBsAg and/or anti-HBc seroconversion incident infection, evidence of vaccination or last visit. Poisson regression was used to determine associated factors. 25,973 had at least one HBsAg test result. Participants with HBsAg results were typically MSM (57%) and white (59%) (similar to the cohort as a whole). The cumulative prevalence of detectable HBsAg was 6.9% (6.6 to 7.2%). Among the 3,379 initially HBV-susceptible patients, the incidence of HBV infection was 1.7 (1.5 to 1.9)/100 person-years. Factors associated with incident infection were older age and IDU. The main limitation of the study was that 30% of participants did not have any HBsAg results available. However baseline characteristics of those with results did not differ from those of the whole cohort. Efforts are on-going to improve data collection.

**Conclusions:**

The prevalence of HBV in UK CHIC is in line with estimates from other studies and low by international standards. Incident infection continued to occur even after entry to the cohort, emphasising the need to ensure early vaccination.

## Introduction

Hepatitis B virus (HBV) is one of the world’s most important infectious diseases, with one third of the world’s population having been infected, approximately 350 million being chronically infected and one million dying of the complications of HBV infection each year [1]. An estimated 33.3 million people worldwide are living with HIV infection and 1.8 million died in 2009 from AIDS-related causes [2]. Due to shared modes of transmission (sexual, blood borne and mother-to-child) HBV and HIV co-infection is common. Co-infection with HBV does not appear to affect the rate of progression of HIV-disease (such as progression to a new AIDS diagnosis) or virological or immunological responses to highly-active antiretroviral therapy (HAART), although there are some conflicting data [3]–[6]. In contrast, HBV outcomes are altered in the setting of HIV. In addition to the higher incidence, there are lower rates of resolution of infection, faster progression of liver disease in those who become chronic carriers, increased rates of adverse drug reactions [7] and increased rates of liver-related death [8]. With the dramatically improved survival of HIV positive individuals with access to HAART [9] liver disease has become one of the most common non-AIDS-related causes of mortality; in the prospective D:A:D cohort liver disease was responsible for 14.5% of deaths, with AIDS causing 31%, cardiovascular disease 11% and non-AIDS cancers 9%. Viral hepatitis is the most important cause of these deaths with 76% occurring in patients with hepatitis B and/or hepatitis C co-infection [10].

Geographical differences exist in HBV incidence, prevalence and genotype so that the quantitative impact of co-infection in the UK cannot be extrapolated simply from international data. Estimates of the prevalence of chronic HBV infection in the general population in the UK are limited. Rates in low-risk HIV-negative groups are low with 0.35% of antenatal samples positive for HBsAg in 2008 [11]. An estimate of the prevalence in the HIV-positive population was made by the EuroSIDA cohort in which 9.1% were co-infected with HBV in northern and central European centres (which include the UK) [4]. Hepatitis B is a preventable disease since an effective vaccine is available. Despite the increased importance of HBV prevention in HIV-positive patients and national policies that recommend vaccination [12], coverage remains incomplete [13], [14].

The UK Collaborative HIV Cohort (UK CHIC) Study collects routine clinical data on patients aged over 16 years attending any of 13 centres for HIV care. It records demographics, clinical events, antiretroviral medication history and laboratory test results, including results of hepatitis B and C serology. The dataset, including data collected up to 2009, contains the records of over 37,000 patients which is over one third of all those diagnosed with HIV in the UK [15]. It thus provides an exceptional resource for examining the epidemiology and impact of HBV co-infection. In this analysis we have used the UK CHIC dataset to estimate the prevalence and incidence of HBV within the UK HIV positive population, and to examine trends over time.

## Methods

Data from the United Kingdom Collaborative HIV Cohort (UK CHIC) Study was used for this analysis. This is an observational cohort of HIV-positive individuals attending some of the largest HIV treatment centres in the UK (see Appendix). Data collected included information on demographics, single most likely route of HIV exposure, antiretroviral history, laboratory test results including HBV serology, AIDS defining events and deaths. Of the 13 centres which provided data to UK CHIC, 12 included HBV data and were included in this analysis. HBV data comprised results of tests for hepatitis B surface antigen (HBsAg), antibody to hepatitis B surface (anti-HBs), and antibody to hepatitis B core (anti-HBc). Hepatitis B DNA results are not available.

The number of individuals under follow up in each year from 1996 to 2009 was determined using first seen and last seen dates and the cumulative proportion of these individuals with HBV data available in each year (up to and including 2009) was calculated. The HBV status for each individual was determined using their last available HBsAg, anti-HBs and anti-HBc result by the end of each year, with results carried forward if not performed, or repeated, in that year. The test result combinations used to define the HBV status of each individual are shown in Table 1; these required a number of assumptions to be made when results were missing or incomplete, and because HBV vaccination records were not available in the dataset. Also, since HBV DNA data is not available, some patients without HBsAg may have been misclassified as uninfected when they in fact had HBsAg negative, DNA positive hepatitis B.

**Table 1:** Hepatitis B result combinations used to define hepatitis B status

		HBsAg	Anti-HBs	Anti-HBc
Not exposed, susceptible		Negative	Negative	Negative
Not exposed, possibly vaccinated		Negative	Missing	Negative
Immune	Vaccinated	Negative	Positive	Negative
	Resolved infection	Negative	Positive	Positive
	Either vaccinated or resolved infection	Negative	Positive	Missing
Isolated anti-HBc		Negative	Negative	Positive
Currently infected		Positive	Any	Any
Unclassifiable (insufficient data)		Negative	Missing	Missing or positive
		Negative	Negative	Missing
		Missing	Any	Any

HBsAg: Hepatitis B surface antigen. Anti-HBs: antibody to hepatitis B surface antigen. Anti-HBc: antibody to hepatitis B core antigen.

Note to Table 1: There are several potential explanations for results being recorded as missing, with varied consequences for the analysis. Tests may have been undertaken but the data not available electronically and therefore not included in the exported dataset, or tests may not have been carried out in error or intentionally. The latter is particularly likely where previous test results are known so that clinicians may have been able to infer the current HBV status, but the details of these are not included in the dataset. In other situations, clinicians or the laboratory may have limited the tests requested, for example if HBsAg and anti-HBc were both detectable an anti-HBs test might have been deemed unnecessary. Some missing results are less critical to the analysis than others, in that the result would not change the allocated category. All those with missing HBsAg results have been put in the unclassifiable category.

The cumulative prevalence of HBV infection was calculated by dividing the number of individuals with a positive HBsAg test result by the number of individuals with an HBsAg test result at any time up to the time period of interest.

Logistic regression was used to assess the association between either having a HBsAg test result in the dataset, or having current HBV infection, and demographic factors (age, ethnicity, risk group), year of cohort entry, CD4 at cohort entry and HIV viral load at cohort entry.

Amongst individuals with a positive HBsAg test result indicating current infection, subsequent HBsAg test results were reviewed in order to further define their HBV status. Individuals were defined as having chronic infection if they had a positive HBsAg test result at least 6 months after their first positive HBsAg test result, with no intervening negative result. Individuals without chronic HBV and with at least one negative HBsAg test result after their first positive HBsAg test result were defined as having had recent infection which resolved. Individuals that did not meet the criteria for either of these categories, for example those with no subsequent HBsAg test results after a single positive HBsAg test result were defined as infected, but not further classifiable.

Associations between the presence of recent HBV infection and demographic and laboratory factors were assessed using logistic regression.

In further analyses, the incidence of HBV infection was calculated. Using all recorded test results within a year of the first HBV test result, individuals were defined as having never been infected with HBV if the result of anti-HBs was negative and the results of HBsAg and anti-HBc were either negative or missing. HBV infection, including both current and resolved infection, was defined as a positive HBsAg result or a positive anti-HBc result. A sensitivity analysis was performed limiting the definition to those with initial negative results for all of HBsAg, anti-HBs and anti-HBc. Incidence rates were calculated for patients who were shown not to have already been infected. Time of infection was defined by the first positive HBsAg or anti-HBc test result, and patients were censored at their last visit or at their first positive anti-HBs result (without a positive anti-HBc result, indicating vaccination and therefore that the patient was deemed no longer susceptible). Rates of HBV infection were determined by dividing the number of newly infected individuals by the number of patient years of follow up, and were stratified by demographic and laboratory variables. Poisson regression was used to assess the associations between incident HBV infection and other factors of interest. All analyses were performed using SAS version 9.

The study obtained ethics approval from the West Midlands Multi-centre Research Ethics Committee (number MREC/00/7/47).

## Results

Of the 37,331 individuals attending centres providing at least some HBV test data, 27,450 (73.5%) had at least one HBV serological test result and 25,973 (69.6%) had at least one HBsAg test result available. Baseline characteristics of these individuals are shown in Table 2. In line with the UK CHIC cohort as a whole, individuals with HBV test results (either any HBV test result [data not shown], or HBsAg specifically) were likely to be men who have sex with men (MSM) and of white ethnicity. At the date of the first test, the median CD4 count was 340 cells/mm^3^, the median HIV viral load was 4.3 log copies/mL and the median age of individuals was 35 years. The proportion of patients under follow up with any HBV data recorded increased from 33% in 1997 to 88% in 2009.

**Table 2:** Baseline characteristics of those with and without HBsAg test results.

All		HBsAg test	No HBsAg test
		25973	11358
Ethnicity N (%)	White	15348 (59.1)	5590 (49.2)
	Black	7438 (28.6)	3447 (30.4)
	Other	3187 (12.3)	2321 (20.4)
Exposure N (%)	MSM	14743 (56.8)	3635 (32.0)
	Heterosexual	7841 (30.2)	3491 (30.7)
	IDU	773 (3.0)	616 (5.4)
	Other	2616 (10.1)	3616 (31.8)
Year of entry N (%)	1996–1999	9323 (35.9)	4743 (41.8)
	2000–2004	8290 (31.9)	2749 (24.2)
	2005–2010	8360 (32.2)	3866 (34.0)
CD4 at first test (cells/mm^3^)	Median (IQR)	340 (173 to 515)	300 (129 to 490)
VL at first test (log copies/ml)	Median (IQR)	4.3 (3.1 to 5.0)	4.1 (2.9 to 4.9)
Age at first test (years)	Median (IQR)	35 (30 to 41)	35 (30 to 41)

HBsAg: Hepatitis B surface antigen. MSM: men who have sex with men. IDU: intravenous drug user. VL: HIV viral load. IQR: interquartile range.

### Predictors of the Availability of HBsAg Test Results

In univariable analyses, patients of white ethnicity and of MSM risk group were more likely to have a HBsAg test result than those of black or other ethnicities and those of non-MSM risk groups (p<0.0001). Individuals were also more likely to have a HBsAg test result if they had higher CD4 counts (OR: 1.02 (95% CI: 1.02 to 1.03) per 50 cells higher) and higher viral loads at entry (1.08 (1.05 to 1.10) per 1 log higher). These variables remained significantly associated with the likelihood of having a HBsAg test result in multivariable analyses.

### Cumulative Prevalence of HBsAg

Of the 25,973 patients with at least one HBsAg test result, 1,781 had at least one positive result, giving a cumulative HBsAg prevalence of 6.9% (6.6% to 7.2%). Factors associated with a positive result amongst all those with HBsAg test data are shown in Table 3. In univariable analyses, those of black and other ethnicities were more likely to have a positive HBsAg test result compared to those of white ethnicity. Heterosexual females were more likely to have a positive HBsAg test result compared to MSM, who were more likely to be positive than heterosexual men. Those who entered the cohort in earlier calendar years were also more likely to have a positive HBsAg test result. No association was seen with HIV viral load or age, although those with higher CD4 counts at their first HBsAg test were less likely to have a positive HBsAg test result. With the exception of the association with gender, all associations with a positive HBsAg test result remained statistically significant in multivariable analyses.

**Table 3:** Associations between factors of interest and positive HBsAg status.

		Univariable		Multivariable	
All		OR (95% CI)	P-value	OR (95% CI)	P-value
Ethnicity	White	1	<0.0001	1	<0.0001
	Black	1.38 (1.24 to 1.54)		2.53 (2.05 to 3.12)	
	Other	1. 25 (1.08 to 1.45)		1.73 (1.39 to 2.14)	
Exposure	MSM	1	<0.0001	1	<0.0001
	Heterosexual (m)	0.75 (0.65 to 0.86)		0.43 (0.34 to 0.55)	
	Heterosexual (f)	1.44 (1.25 to 1.65)		0.87 (0.69 to 1.09)	
	IDU	1.22 (0.93 to 1.59)		1.45 (0.95 to 2.20)	
	Other	1.03 (0.88 to 1.22)		0.66 (0.51 to 0.86)	
Year of entry	1996–1999	1.73 (1.53 to 1.95)	<0.0001	1.74 (1.44 to 2.11)	<0.0001
	2000–2004	1.40 (1.23 to 1.60)		1.29 (1.10 to 1.50)	
	2005–2010	1		1	
CD4 at CHIC entry (cells/mm^3^)	Per 50 cells higher	0.94 (0.93 to 0.96)	<0.0001	0.94 (0.93 to 0.96)	<0.0001
VL at CHIC entry (log copies/ml)	Per 1 log higher	1.01 (0.96 to 1.07)	0.59	0.93 (0.88 to 0.98)	0.01
Age at first test (years)	Per 10 years older	1.00 (0.95 to 1.06)	0.90	1.01 (0.93 to 1.09)	0.88

OR: odds ratio. CI: confidence interval. MSM: men who have sex with men. IDU: intravenous drug user. VL: HIV viral load.

### Resolution vs Chronicity

Of the 1,781 individuals who had ever had a positive HBsAg test result, 758 patients could not be further classified as having recent or chronic infection from the data available. Of the remaining 1,023 individuals, 836 (81.7%) had chronic hepatitis B, and 187 (18.3%) had recent infection which resolved. In univariable analyses, patients with resolution of HBV were more likely than those with chronic HBV infection to be of white ethnicity (OR 0.45 (0.30 to 0.68) and 0.73 (0.43 to 1.26) comparing black and other ethnicity to white ethnicity) and had higher HIV viral loads at the time of their first positive HBsAg test result (1.15 (1.01 to 1.32)). There were no differences between exposure groups. After adjusting for potential confounders, only ethnicity remained significantly associated with resolved HBV infection (0.41 (0.25 to 0.67) and 0.66 (0.34 to 1.28) comparing black and other ethnicity to white ethnicity).

### Change in HBV Prevalence Over Time

Of the 2698 patients who had HBV test results recorded before the end of 1996, the first year of the cohort, 185 (6.9%) had a positive HBsAg result. In 2009, amongst the 22,734 patients under follow up in that year, 19,953 had at least one HBV test recorded and 1027 (5.1%)of these patients had a positive HBsAg test result. Nine patients in 1996 and 25 in 2009 were positive for both HBsAg and anti-HBS and although simultaneous positive HBsAg and anti-HBs has been described [16] it is possible that these results represented data errors. However excluding these did not significantly change the prevalence estimations; 6.5% (176/2698) in 1996 and 5.0% (1002/22,734) in 2009.

**Figure 1:**
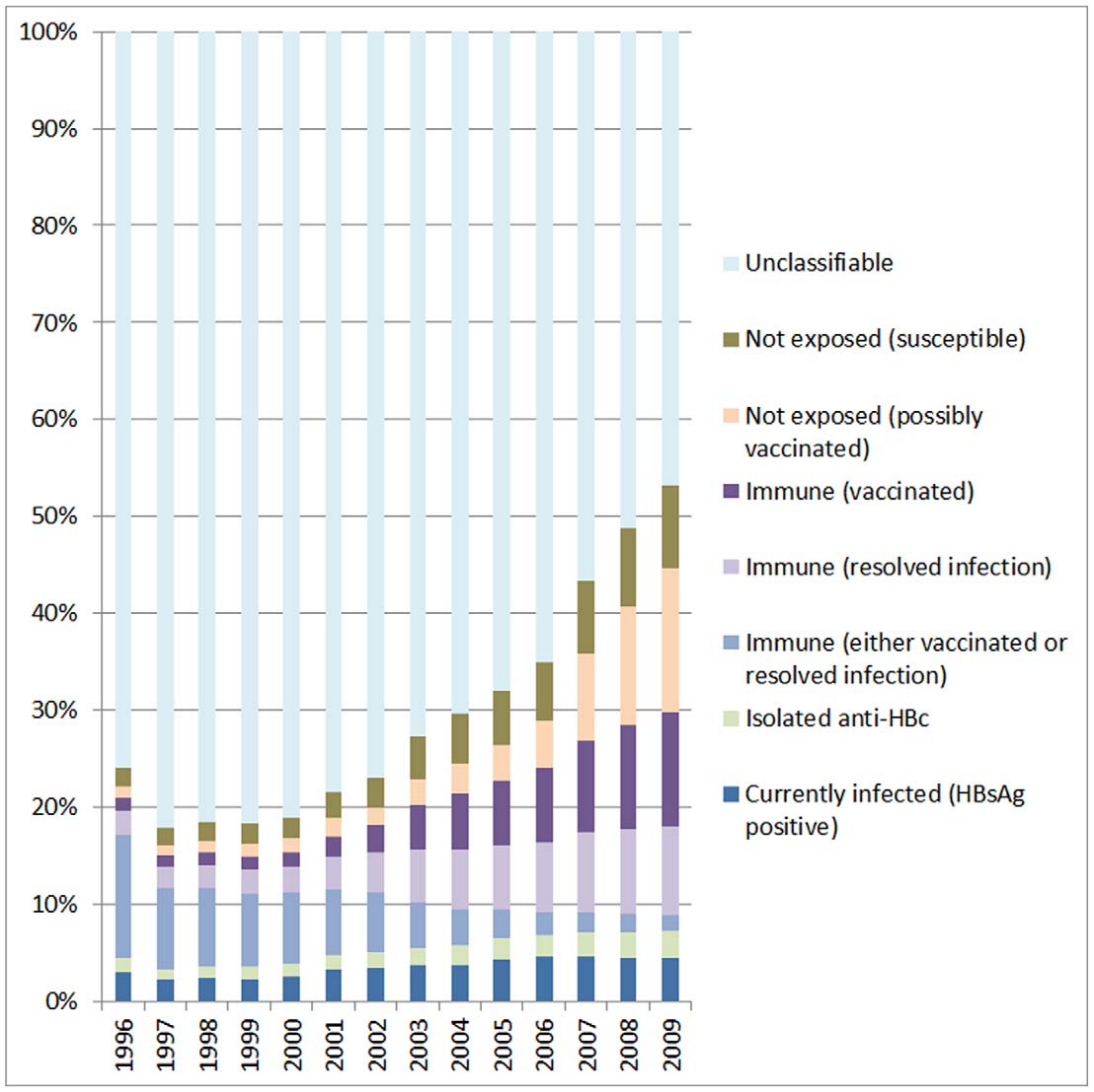
Hepatitis B status by calendar year. Proportion of patients with current or resolved HBV infection, never exposed to HBV and with unclassifiable HBV status, stratified by calendar year.

Considering the whole cohort (including those without hepatitis B test results), current infection (defined as HBsAg positive) was present in 3.0% of those seen for follow-up in 1996 and 4.5% of those seen in 2009. The proportion of patients defined as immune as a result of prior infection (resolved infection) increased from 2.5% in 1996 to 9.0% in 2009 and the proportion with isolated anti-HBc increased from 1.5% to 2.8% over the same time period. Combining these, gives an overall proportion of patients with evidence of ever having been infected with HBV of 7.0% in 1996 increasing to 16.4% in 2009 (figure 1).

### Incident HBV Infection

3379 patients could be categorised as susceptible to HBV infection and subsequently contributed a total of 15,001 person-years of follow up with serological test results available. Of these, 252 had a positive HBsAg or anti-HBc test result during the period of observation, giving in an overall HBV incidence rate of 1.7 (1.5 to 1.9)/100 person-years. Table 4 shows the events, person years of observation and associations with HBV incidence. HBV exposure rates were highest in intravenous drug users (IDU), then MSM, and lowest in heterosexuals. Individuals of older age and those with prior AIDS diagnoses were more likely to acquire HBV, but after adjusting for potential confounders, only risk group remained significantly associated with incident infection. Further test results were available for 200 patients after incident HBV and of these 33 or 16.5% (11.6 to 22.4%) were HBsAg positive for at least six months indicating that they had failed to resolve the infection and had become chronically infected. A sensitivity analysis, in which the definition of susceptible was restricted to those with negative HBsAg, anti-HBs and anti-HBc results, found 45 events in 1,837 patients with 6,476 person-years of follow-up, The incidence of HBV was thus 0.7 (0.5 to 0.9)/100 person-years. Subsequent results were available for 36 of the 45 and 33.3% met the definition of chronic infection.

**Table 4:** Associations between demographic, laboratory and clinical characteristics and incidence of hepatitis B.

			Univariable		Multivariable	
All		Events/person-years	RR (95% CI)	P-value	RR (95% CI)	P-value
Ethnicity	White	185/10553	1	0.37	–	
	Black	50/3093	0.92 (0.67 to 1.26)			
	Other	17/1355	0.72 (0.44 to 1.18)			
Risk group	MSM	183/9191	1	<0.0001	1	<0.0001
	Heterosexual (m)	19/1542	0.62 (0.39 to 0.99)		0.59 (0.36 to 0.94)	
	Heterosexual (f)	20/2321	0.43 (0.27 to 0.69)		0.44 (0.28 to 0.70)	
	IDU	14/409	1.72 (1.00 to 2.96)		1.70 (0.98 to 2.92)	
	Other	16/1541	0.52 (0.31 to 0.87)		0.53 (0.32 to 0.89)	
Current VL	<50	99/5800	1	0.98	–	
(copies/ml)	>50	126/7558	1.02 (0.79 to 1.33)			
	Missing	27/1643	0.99 (0.65 to 1.49)			
Age at first test	<35	70/5327	1	0.03	1	0.06
(years)	35–45	122/6396	1.45 (1.08 to 1.95)		1.40 (1.04 to 1.89)	
	>45	60/3278	1.39 (0.99 to 1.97)		1.37 (0.96 to 1.95)	
Previous AIDS	No	190/12114	1	0.04	1	0.10
	Yes	62/2888	1.37 (1.03 to 1.82)		1.28 (0.96 to 1.72)	
Current CD4	<200	35/1919	1.19 (0.79 to 1.78)	0.58	–	
(cells/mm^3^)	201–350	57/3496	1.06 (0.75 to 1.51)			
	351–500	70/3614	1.26 (0.91 to 1.76)			
	>500	71/4628	1			
	Missing	19/1345	0.92 (0.55 to 1.53)			
Current calendar year	1996–1999	34/2015	1.20 (0.81 to 1.79)	0.17	–	
	2000–2002	56/3002	1.33 (0.95 to 1.87)			
	2003–2005	79/4063	1.39 (1.02 to 1.89)			
	>2005	83/5921	1			

RR: relative risk. CI: confidence interval. MSM: men who have sex with men. IDU: intravenous drug user. VL: HIV viral load.

### ‘Not infected’ groups

Individuals were defined as ‘not infected’ if they fell into one of the following 3 groups: not exposed and susceptible; not exposed but possibly vaccinated and immune; and vaccinated (Table 1). Figure 1 shows trends over time for these groups, where the denominator is all patients in the cohort (including those without test data). Using the last known status of patients in each year, the proportion of patients who were susceptible to HBV increased from 1.9% in 1996 to 8.6% in 2009. The proportion of patients who were not exposed but possibly vaccinated increased from 1.2% in 1996 to 14.8% in 2009 and the proportion of patients who were immune due to vaccination increased from 1.4% in 1996 to 11.9% in 2009. The overall proportion of patients who were not infected with HBV increased from 4.4% in 1996 to 35.2% in 2009.

### Vaccination Status

Due to incomplete results, whether through lack of testing or incomplete data capture, it is not possible to determine precisely the number vaccinated and the number susceptible. Data on vaccination history itself was not available as part of the UK CHIC dataset. However if the analysis is limited to those with sufficient test results available, it is possible to estimate the coverage of vaccination among those who have not already been infected given by the number with the serological pattern of a vaccine responder (detectable anti-HBs in the absence of anti-HBc, and HBsAg) as a proportion of the sum of this group and those classified as “never infected (susceptible)”. This increased from 42.0% in 1996 to 58.2% in 2009 (p<0.001, Chi squared).

## Discussion

The cumulative prevalence of current HBV infection (HBsAg positivity) in the UK CHIC cohort was 6.9% (6.6 to 7.2%). The prevalence amongst those under follow-up and tested in 2009 was 5.1%. This is lower than the regional estimate of 9.1% from EuroSIDA (northern and central Europe: UK, Eire, Norway, Sweden, Denmark, Germany, Netherlands, Belgium, Luxembourg, France and Switzerland). The prevalence of HBV infection is related to patient characteristics including mode of HIV transmission and place of birth (and hence the association with ethnicity in this population). Of note we found that those of black or other ethnicity and those with a history of IDU had a higher prevalence while heterosexual men and women had lower prevalence, when compared to MSM. Those who entered the cohort earlier were more likely to have had a positive HBsAg test result at some time during follow up. However this difference may reflect longer follow up rather than a true decline in prevalence over time. They were also less likely to have had HBV vaccination or to have contracted HBV before HIV. The completeness of data capture, which was lower earlier in the period of the UK CHIC study could also have resulted in some bias; for example HBsAg positive patients may have been more likely to have been retested and so included in the dataset.

Using the last available HBsAg result for each individual, there was a declining prevalence of current HBV infection from 6.9% in 1996 to 5.1% in 2009. This may be due to changes in demographics, with fewer patients coming from, for example, areas of high HBV prevalence in sub-Saharan Africa, or may indicate better vaccination coverage and hence prevention of infection. It may also be attributed to the marked increase in the proportion of the cohort for which HBV status could be fully defined, associated, with more complete data capture, with or without more comprehensive testing in clinics. Of those with current HBV infection for whom there was sufficient data, the majority (81.7%) were HBsAg-positive for at least six months, meeting the definition of chronic hepatitis B. Some patients who lost detectable HBsAg within 6 months could have been late chronic infections, but the rate of HBsAg loss in those with chronic infection is very low so that misclassifications will be rare [17]. HIV infection is associated with a lower rate of clearance after acute HBV infection, with one fifth likely to proceed to chronic hepatitis [18], [19]. Since the majority of HIV infections in black patients in the UK were acquired in Africa [20] it is likely that most HBV infections in non-white individuals in this cohort will have been acquired prior to HIV infection, either by vertical or early childhood infection, which predominates in endemic areas. Most white patients were either MSM or IDU, and therefore likely to have only been at risk of HBV infection as adults. The higher proportion of resolved infections amongst the white ethnic group is therefore consistent with the lower risk of chronicity in adults, even in those who are HIV positive, compared to that seen in vertical or early childhood acquired infection.

The proportion of those never infected with HBV who were protected by vaccine increased between 1996 and 2009 (from 42.0% to 58.2%). This may be related both to an improvement in vaccine coverage and to improved vaccine response rates as the mean CD4 count of the cohort has increased. Nonetheless, around 40% of those not already infected remained at risk of HBV infection at the time of the last available test result. In the absence of vaccination data we cannot be certain how much of this is due to failure to vaccinate, and how much to the impaired immune response to vaccine in HIV positive individuals. National guidelines continue to recommend vaccination for all HIV-positive patients at diagnosis, with a repeat course in non-responders. Repeating the course when CD4 counts have increased on treatment may also be worthwhile. Regular review or clinical audit, as recommended by the British HIV Association, should enable providers to monitor their service performance and adjust clinic practice accordingly [12].

Whether due to failure to vaccinate, or inadequate response, we found evidence of incident HBV infection occurring in the cohort at a rate of 1.7 cases per 100 person-years of follow up, though this figure must be interpreted with caution in view of the lower value of 0.7 cases per 100 person-years found in the sensitivity analysis. Infection frequently gave rise to chronic hepatitis B, in 16.5% of cases, which is consistent with published data [18], [19]. The risk of incident HBV infection was higher for IDU than for MSM and higher for MSM than for heterosexuals, perhaps reflecting poorer vaccination coverage in the former group and lower ongoing risk in the latter. Of note older patients had a higher risk of new HBV infection, demonstrating the continuing risk of sexually transmitted infection in this group, possibly combined with failure of vaccine protection over time.

Overall the proportion of patients in UK CHIC with any HBV results available was just under 75% with about 70% having at least one result for HBsAg. UK national guidelines recommend that all patients should have HBV tests at HIV diagnosis, and have their status re-checked annually [12]. Data is submitted to UK CHIC electronically from routine clinic systems and databases. As clinical information systems in use in treatment centres have developed, particularly with integration with laboratory systems, completeness of data has increased over time. Efforts are ongoing to audit and improve data extraction and collection. The EuroSIDA study also uses electronic databases but is supplemented by manually collected data from paper records, which could result in less missing data. Despite this, at the time of estimating HBV prevalence, EuroSIDA contained HBsAg results on only 5728 patients (58.4% of all patients in the study) from a total of 29 countries [4]. Published data from EuroSIDA also included only a single HBsAg result from each patient without other HBV serology and has been reported only at the regional level (with the UK included in the northern and central Europe region). Thus we believe the current study provides a more detailed picture of HBV co-infection in the UK than has been possible before, estimating not only HBV prevalence but also incidence and the proportion still at risk of infection.
